# A Simplified Irrigation Pump Testing Method for Developing Countries: A Case Study in Bangladesh†

**DOI:** 10.1002/ird.2269

**Published:** 2018-07-31

**Authors:** Su Yu, Jonathan S. Colton, Md. Abdul Matin, Timothy J. Krupnik

**Affiliations:** ^1^ George W. Woodruff School of Mechanical Engineering Georgia Institute of Technology Atlanta Georgia USA; ^2^ Farm Machinery and Postharvest Process Engineering Division Bangladesh Agricultural Research Institute Joydebpur Gazipur Bangladesh; ^3^ International Maize and Wheat Improvement Centre Dhaka Bangladesh

**Keywords:** axial‐flow pump, small‐scale farmers, manufacturing analysis, computer‐aided engineering, numerical simulation, full‐scale field test, pompe à flux axial, petits agriculteurs, analyse de fabrication, ingénierie assistée par ordinateur, simulation numérique, essai sur le terrain à grande échelle

## Abstract

Much of South Asia experiences a monomodal rainfall pattern with a distinct dry season following the annual monsoon. Enabling irrigation during the dry season has therefore been crucial in assuring improved productivity and double‐cropping. This is particularly the case in southern Bangladesh, where recent government initiatives have called for an expansion of surface water irrigation to reduce pressure on groundwater tables in intensively cultivated areas in the north of the country, where dry season *boro* rice is grown. This paper describes a method based on first principles of fluid mechanics to characterize the performance of surface water irrigation pumps used by small‐scale farmers in South Asia and Bangladesh. This method is unique, as it incorporates an optimized protocol suitable for resource‐limited conditions found in many developing countries and provides a comprehensive yet simple‐to‐use pump selection method for surface water irrigation pump customers. Using pump impellers as a case study, the method also characterizes the effect of pump geometric variations resulting from the variable production and assembly practices found in different manufacturing workshops. This method was validated with a case study in Bangladesh supported by both full‐scale field testing and numerical simulation results. © 2018 The Authors. Irrigation and Drainage published by John Wiley & Sons Ltd on behalf of International Commission for Irrigation and Drainage

## Résumé

Une grande partie de l'Asie du Sud connaît un régime pluviométrique monomodal avec une saison sèche distincte après la mousson annuelle. Permettre l'irrigation pendant la saison sèche a donc été crucial pour assurer une meilleure productivité et une double culture. C'est particulièrement le cas dans le sud du Bangladesh, où des initiatives gouvernementales récentes ont demandé une expansion de l'irrigation avec des eaux de surface pour réduire la pression sur les nappes phréatiques dans les zones intensément cultivées du nord du pays où pousse le riz *boro* de saison sèche. Cet article décrit une méthode basée sur les premiers principes de la mécanique des fluides pour caractériser les performances des pompes d'irrigation avec des eaux de surface utilisées par les petits agriculteurs en Asie du Sud et au Bangladesh. Cette méthode est unique car elle incorpore un protocole optimisé adapté aux conditions de ressources limitées dans de nombreux pays en développement et fournit une méthode de sélection de pompe complète mais simple à utiliser pour les clients de pompes d'irrigation d'eau de surface. En utilisant des turbines de pompage comme étude de cas, la méthode caractérise également l'effet des variations géométriques de la pompe résultant des pratiques variables de production et d'assemblage trouvées dans différents ateliers de fabrication. Cette méthode a été validée à l'aide d'une étude de cas au Bangladesh appuyée à la fois par des essais sur le terrain à grande échelle et des résultats de simulation numérique. © 2018 The Authors. Irrigation and Drainage published by John Wiley & Sons Ltd on behalf of International Commission for Irrigation and Drainage

## Introduction

The country of Bangladesh has a total area that is just 5% of India, but with a population that exceeds 165 million people (Schwartzberg 2016). Bangladesh, however, produces over one‐third the amount of rice that is produced in India, and in most years manages rice self‐sufficiency (Bangladesh Agricultural Research Corporation 2013; Food and Agriculture Organization of the United Nations (FAO) 2014; Mainuddin and Kirby 2015). This productivity was enabled largely through the expansion of groundwater irrigation for dry season *boro* rice production in the country's northern plains (Qureshi *et al*. 2015). Concerns regarding the environmental sustainability of groundwater abstraction in these intensively irrigated areas—particularly in consideration of the high energey costs required to vertically lift water from aquifers and regional groundwater overdraft (McBean *et al*. 2011; Shamsudduha *et al*. 2011; Qureshi *et al*. 2014, 2015; Kirby *et al*. 2015; Shahid *et al*. 2015)—have caused policy makers to propose increased use of available fresh surface water resources for irrigation in southern Bangladesh (Ministry of Agriculture (MoA) and FAO 2013).

The challenge, however, is to make effective use of available surface water resources for double‐cropping while using affordable and energy‐efficient pump sets, typically powered by diesel (Krupnik *et al*. 2015; Pradeleix *et al*. 2015). One option to address these goals is to replace high‐fuel‐demanding centrifugal pumps with more energy‐efficient axial‐ and mixed‐flow pumps where surface water lift requirements are less than 3 m in height (International Rice Research Institute 1983; Karassik *et al*. 2007; Ministry of Power 2013), as is the case in much of southern Bangladesh (Krupnik *et al*. 2017). In a recent comparison with centrifugal pumps, Krupnik *et al*. (2015) found that axial‐ and mixed‐flow pumps can increase energy efficiency by 51 and 21% at 1 m and 2 m heads respectively, corresponding to an economic saving of US$38–70 ha^‐1^ season^‐1^ resulting from significantly lower fuel and irrigation time requirements for dry season *boro* rice production.

Use of axial‐ and mixed‐flow pumps has increased in Bangladesh and South Asia, with manufacturers showing increased interest in this pump technology (CSISA‐MI 2016). Machinery design and manufacturing abilities in Bangladesh and in many developing countries are often limited, with non‐standard production line processes and little to zero post‐production product testing or quality assurance (Krupnik *et al*. 2013). Therefore, this paper focuses on the design and manufacture of axial‐ and mixed‐flow pumps. This paper proceeds from previous work on the parametric design phase of irrigation pump design and optimization (Yu and Colton 2017), and presents a simple method for testing irrigation pumps that allows farmers and service providers to more easily select and operate irrigation pumps in the most economical conditions. Although it focuses on Bangladesh, the engineering principles and processes detailed in this paper are broadly applicable to other developing country circumstances, such as sub‐Saharan Africa and other South Asia countries, where irrigation pump manufacturers may have limited resources for quality control and product testing (Molle *et al*. 2003; Knox *et al*. 2013; Kamwamba‐Mtethiwa *et al*. 2016).

## Technical Specifications of Irrigation Pumps

### Specific speed and pump classification

Rotodynamic pumps, machines with a wheel or rotor whose rotating blades or vanes impart tangential acceleration to a liquid flow, are categorized into three types based on flow direction (Addison 1955). Examples include axial‐flow pumps (AFPs) that generate axial flow, radial‐flow pumps or centrifugal pumps (CPs) that generate radial flow, and mixed‐flow pumps (MFPs) that generate both types of flow (Wilcox 2000). Their application range is described by their specific speed in dimensional form as *N*_sd_, as shown in Equation (1):

(1) $$N_{\mathrm{sd}}=\frac{\Omega \sqrt{Q}}{{\left(H\right)}^{3/4}}$$

where *Ω* is the pump shaft speed (rpm), *Q* the volumetric flow rate (m^3^ h^‐1^) and *H* the pressure head (m). Physically, the specific speed is the operating speed at which a pump achieves unit head at a unit volume of flow rate. Low values ( $N_{\mathrm{sd}\left(m^{3}h^{-1},m\right)}$ < 4650) correspond to efficient CP operation. Moderate values correspond to MFPs, whereas high values ( $N_{\mathrm{sd}\left(m^{3}h^{-1},m\right)}$ > 10 500) correspond to AFPs (Wilcox 2000). Based on these values, AFPs and MFPs are more effective for surface water irrigation at low *H* heads, and therefore would be more suitable than CPs for surface water pumping from canals and rivers in South and South East Asia (Santos Valle *et al*. 2014).

### The friction loss model

To achieve accurate flow measurements, it is necessary to account for friction losses in pipe flow systems because they reduce the discharge by a factor of four. Friction losses consist of: (i) major losses due to viscous effects in straight pipes; (ii) minor losses due to geometry of pipe components (Munson *et al*. 2009). Assuming a steady incompressible flow with a pipe of constant diameter, friction loss is defined by Equation (2) as

(2) $$h_{L}=h_{L\;\text{major}}+h_{L\;\text{minor}}=f\frac{l}{D}\frac{V^{2}}{2g}+K_{L}\frac{V^{2}}{2g}$$

where *l* is the length of the pipe, *D* is the diameter of the pipe, *V* is the velocity of the fluid, *g* is the gravitation constant, f=ϕReϵ/D is the friction factor that is determined from the Moody chart using the Reynolds number, Re; ϵ/D is the relative roughness; and *K*_L_ is the dimensionless minor loss coefficient obtained from references (Moody 1944; Wilcox 2000; Munson *et al*. 2009). As *f*, *l* and *D* remain constant in this paper, $K_{\text{majo}r}=f\left(l/D\right)$ and *K*_minor_ *= K*_L_, Equation (3) is obtained:

(3) $$h_{L}=\left(K_{\text{major}}+K_{\text{minor}}\right)\frac{V^{2}}{2g}$$

Using this friction loss model, the modified Bernoulli equation (Equation (4)), also known as the steady one‐dimensional energy equation, can be used to characterize the flow profiles across pump types:

(4) $${\left(\frac{V^{2}}{2g}+z+\frac{p}{\mathrm{\rho g}}\right)}_{1}-{\left(\frac{V^{2}}{2g}+z+\frac{p}{\mathrm{\rho g}}\right)}_{2}=h_{L}-\frac{w_{p}}{g}$$

where the subscripts (1) and (2) represent two different locations, for example the surface of the irrigation ditch and the exit to the pump, *z* is the elevation head, p/ρg is the pressure head and $-w_{p}/g$ is the pump head.

The loss factor, *K*, can be calculated from measured pressure head, H=p/ρg, at shut‐off valve position (or shut‐off head) and velocity, *V*, at maximum discharge, as Equation (5), which is derived from Equations (3) and (4):

(5) $$K=K_{\text{major}}+K_{\text{minor}}=\frac{H^{2}g}{V^{2}}-1$$

This friction loss model will be applied to assess performance of different pump systems with customized components.

### Dimensional analysis and affinity law

Dimensional analysis (DA) reveals functional relationships among relevant physical quantities such as discharge *Q*, pump shaft speed *N* or diameter *D*, and establishes dimensionless criteria of flow under dynamically similar conditions. DA is widely used to evaluate factors affecting flow (White 2016).

By selecting *E*, *D* and *ρ* as the three independent parameters, where *E = gH* and is the energy applied to the pump shaft and *ρ* is fluid density, and including the Reynolds number, specific speed and specific head, the affinity law, which relates the volumetric flow rates, *Q*, to the rotational speed of the pumps, *N*, and their diameters, *D,* can be expressed as Equation (6) (Stepanoff, 1957):

(6) $$\frac{Q_{1}}{Q_{2}}=\frac{N_{1}}{N_{2}}\;{\left(\frac{D_{1}}{D_{2}}\right)}^{3}$$

Similarly, Equation (7) relates head, *H,* to speed and diameter:

(7) $$\frac{H_{1}}{H_{2}}={\left(\frac{N_{1}D_{1}}{N_{2}D_{2}}\right)}^{2}$$

## Materials and Methods

To assist in the development and testing of surface water irrigation pumps, a standardized and practical testing method is developed for these regions to determine how practical changes in pump design and management impact the local irrigation in a cost‐effective, energy‐efficient manner. Through standard test procedures that accurately measure key variables such as pumping head, water discharge and fuel consumption, this method effectively and reliably assesses pump performance. It is expected that its use can improve the quality of pump design and manufacture in South Asia.

The test method detailed in this paper innovates in three ways. First, the method simulates testing at different head levels using shut‐off valve positions (open, closed or in‐between) on a new instrument designed to reduce test procedure costs and increase the ease of pump evaluation. Second, the method includes numerical testing of multiple pump prototypes using computer simulation in order to increase manufacturing capability while also reducing costs. Finally, the method incorporates friction losses and affinity law theories from fluid mechanics to develop a pump selection method for local customers. This straightforward and easy‐to‐use pump selection method serves as an alternative to traditional pump curves to help farmers with pump purchase and operation.

### Pump testing method

**Figure 1:**
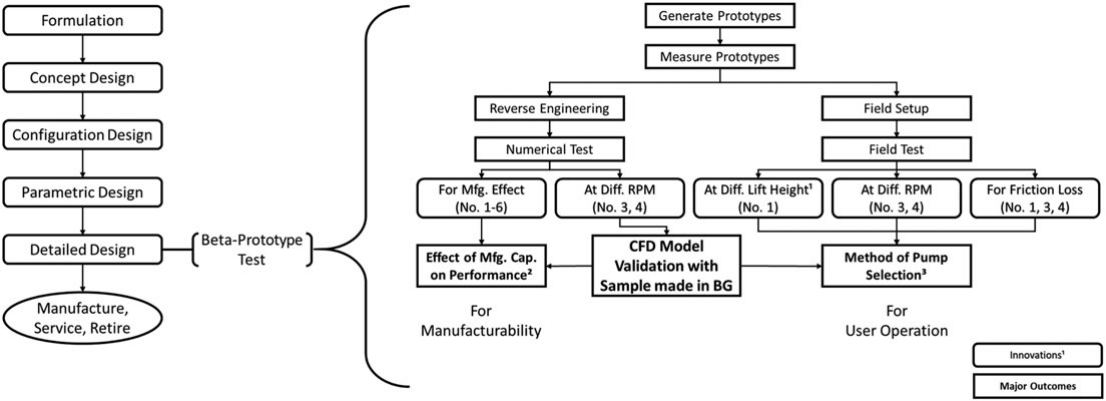
Flow diagram outlining the design and testing procedure.

The design and testing process detailed in this paper is described in Figure 1.

### Testing procedure

**Figure 2:**
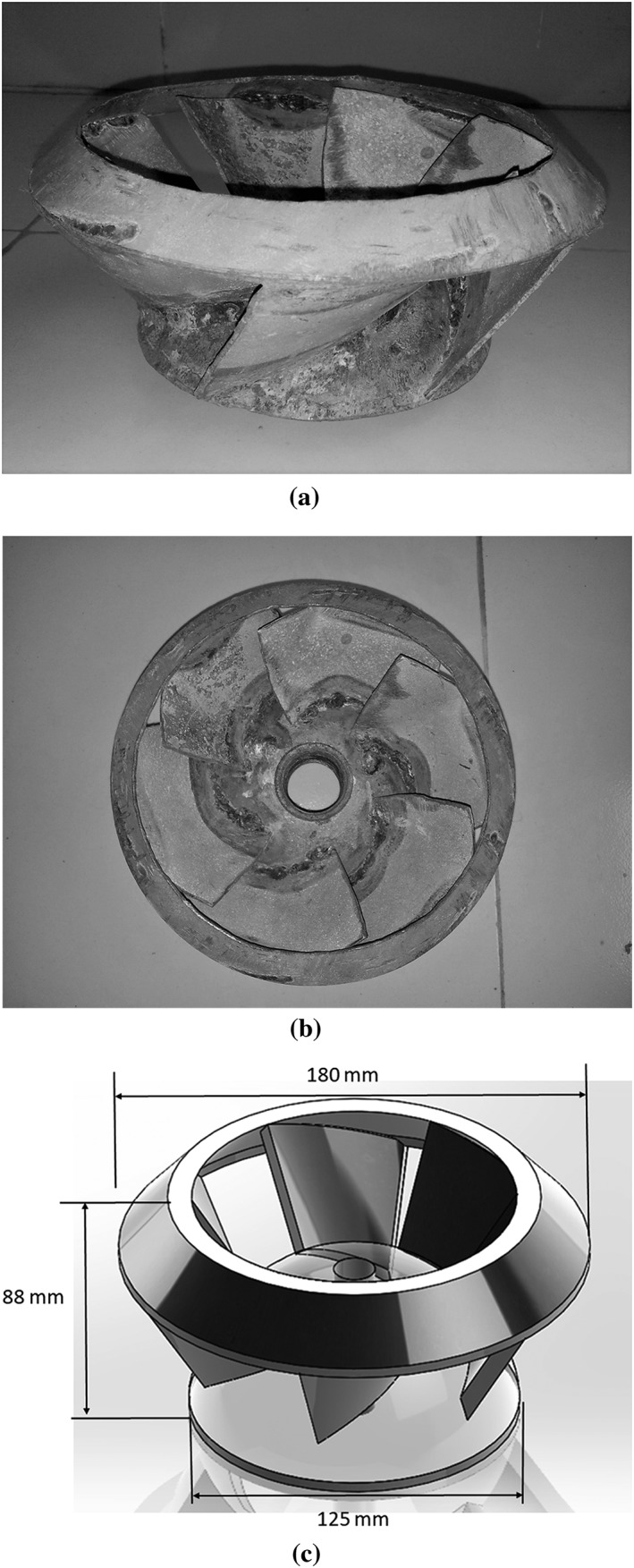
Locally manufactured MFP impeller prototype (a: side view; b: top view) and its CAD model (c). The impellers have six vanes.

Experiments were performed to evaluate the performance and effectiveness of MFPs manufactured locally in Bangladesh, especially their impellers. A set of six full‐scale (150 mm diameter) mild steel sheet pump impellers of the same nominal design and geometry as a commercial Thai MFP impeller were tested (Figure 2).

In this study, pump testing included both field testing and numerical testing through computational fluid dynamics (CFD) simulation. Field test data were used to characterize the accuracy of the theoretical models introduced above, and numerical simulation data to characterize the effect of improving the manufacturing quality of impellers on pump performance. A pump selection method developed from these models is subsequently described.

### Field testing

**Figure 3:**
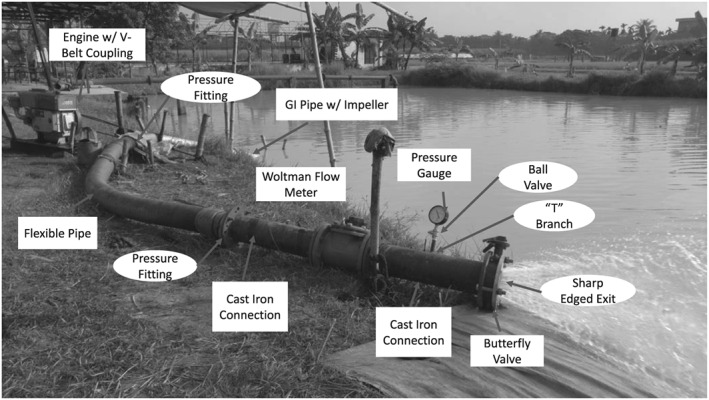
Field test apparatus for component identification and friction loss estimation (adapted from Krupnik *et al*. 2015).

**Figure 4:**
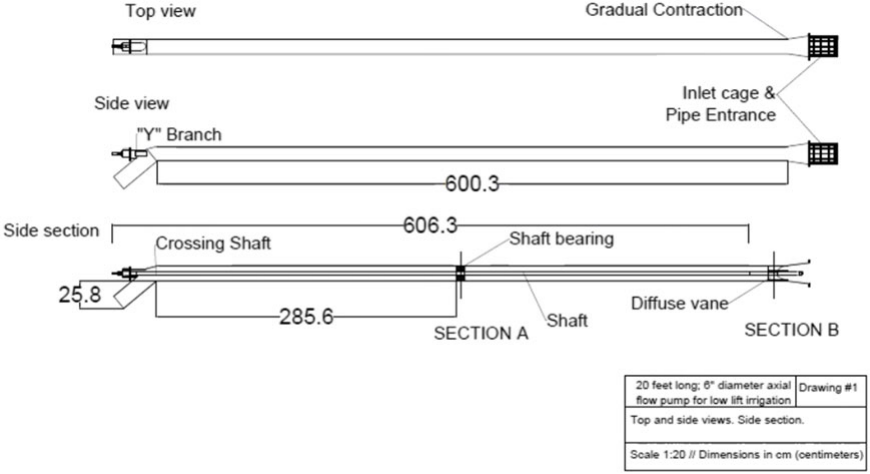
Schematic of GI pipe interior of the MFP (top: top view; middle: side view; bottom: side section view).

Field testing was performed at the Bangladesh Agricultural Research Institute in Gazipur, Bangladesh (23°59′13″ N, 90°24′51″ E) using the testing protocol and pump test bed reported by Lam *et al*. (2017). The test apparatus included both pump and control‐measurement components (Figure 3). The pump component was composed of a 150‐mm diameter pump impeller manufactured by Rahman Engineering (Dhaka, Bangladesh), a galvanized‐iron (GI) pipe of 150 mm diameter and 6 m length, a drive shaft, a V‐belt pulley power coupling, a 12.5 HP Changchai S195 diesel engine (Chagzhou, China), a weighing scale, and an external fuel tank (Santos Valle *et al*. 2014). The cross section of the GI pipe is shown in Figure 4. The impeller was attached to the shaft end and placed at the GI pipe inlet and submerged in pond water as described by Krupnik *et al*. (2015). A shaft bushing and a diffuser vane were located within the pipe to centre the shaft with a cage placed at the inlet to filter debris. The inlet of the pipe was slightly enlarged to accommodate the 180‐mm diameter impeller, with an outlet bent at 45°. The engine drove the shaft at various testing RPMs through the V‐belt coupling (Santos Valle *et al*. 2014).

The control measurement section included a flexible hose, two segments of cast iron pipes, a Woltman flow meter built by Bermad Irrigation (model WPH‐150) (Bermad Irrigation 2013), a pressure gauge and a butterfly control valve (Figure 3) as described by Lam *et al*. (2017). All components except the pressure gauge measured 150 mm nominal diameter.

During the test, the butterfly valve controlled the flow rate and a pressure gauge measured the corresponding pressure (kg cm^‐2^), which was later converted into water head (m). After each test run, the elapsed time was recorded using a stopwatch and total water discharge was measured using the flow meter.

This contrasts with direct approaches that elevate a pump's outlet to study its performance at a new lift height, as described by Krupnik *et al*. (2015) and Santos Valle *et al*. (2014); here, different lift heights are simulated by varying the control valve's position. This approach saves cost and effort and is accomplished by relating the change in friction head to the change in lift height as described in Equation (8):

(8) $$H_{\mathrm{est}}-H=\Delta K\frac{{V_{2}^{*}}^{2}}{2g}$$

where *H*_est_ is the new theoretical lift height with a corresponding flow velocity $V_{2}^{*}$ and Δ *K* is the change of minor loss coefficient of the control valve from its nominal position. As the change in friction head is not measured directly, but rather through the pressure gauge reading, Equation (8) can be alternatively shown to be Equation (9):

(9) $$H_{\mathrm{est}}-H=\frac{P^{*}-P}{\mathrm{\rho g}}$$

where *P* ^*^ is the measured pressure reading from a new valve position corresponding to *H*_est_. This correlates the theoretical lift height with the pressure reading at different valve positions. The relationship between flow velocity and theoretical lift can then be expressed as Equation (10):

(10) $$\frac{K+1}{2g}{V_{2}^{*}}^{2}+H_{\mathrm{est}}=\frac{w_{p}}{g}$$

Assuming constant *w*_*p*_, a linear relationship between theoretical lift height and the velocity squared with a slope of $\left(-K+1/2g\right)$ can be derived.

### Numerical simulations

Nine design parameters—vane width, cone height, impeller height, distance between tops of vanes, distance between edges of vanes, hole diameter, cone diameter, outer diameter and weight—were measured on each of the six Bangladesh manufactured impeller prototypes (Yu 2017). Measurements were subsequently used to create SolidWorks (SolidWorks 2016 Education Edition SP4.0, Waltham, Mass.) CAD models, which were contrasted with manufactured impellers (Figure 2).

**Figure 5:**
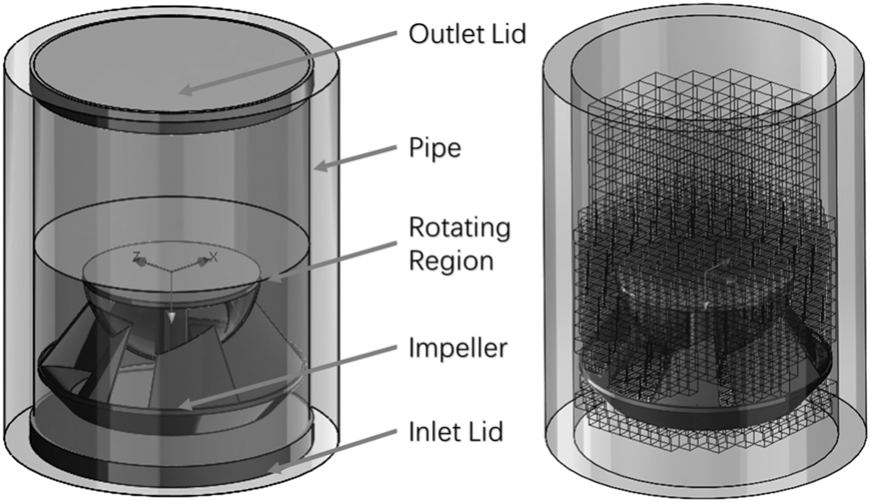
Geometry modelling (left) and mesh profile (right) of CFD model.

A CFD model was generated using SolidWorks Flow Simulation. The model was constructed in three sequential stages: geometric modelling, mesh generation and problem conditions. In the initial stage, the impeller was placed in a pipe segment, which was represented by a thin cylindrical shell with an inner diameter of 187 mm and a length of 270 mm (Figure 5). An optimum clearance of 4 mm is expected between the outer diameter of the impeller and the inner wall of the pipe, as suggested by Aban (1985). Details of the model can be found in Yu (2017).

A grid independence study was performed to validate the accuracy and reliability of the model's results, details of which can be found in Yu (2017). A grid size was selected that provided an accurate result that was insensitive to variations in grid size.

### Pump model evaluation

The effect of geometric variation is quantified through the operating shut‐off head of each impeller. Because the friction losses of the components within the system other than the pump itself are not modelled, calculating the volumetric flow rate from simulations does not yield accurate data per Equation (2). Therefore, shut‐off heads were compared, which are theoretically independent of friction losses.

The friction loss model estimated the pump performance based upon the concepts of major and minor losses introduced above. This model was evaluated by estimating friction loss factors of each component within the system in Figures 3 and 4 from their material and geometry (Crane Co. Engineering Division 2012), and comparing the estimates with the results of the full‐scale experiments.

The affinity law model correlates pump performance at different rotational speeds. As shown in Equations (6) and (7), the discharge rate, *Q*, is expected to be directly proportional to the pump rotational speed, *N*, and the head, *H*, being proportional to *N* ^2^. Therefore, this model was evaluated by fitting first‐ and second‐order lines. Both lines were forced through the origin, as discharge and head were zero when the shaft was stationary.

The lift height model introduced above simulates different lift heights by varying the control valve's position. It is evaluated by fitting experimental data to Equation (10). Based on the assumption of constant pump head, $w_{p}/g$, a linear relationship is expected between theoretical lift height and the velocity squared.

## Results

The results for the CFD approach are validated by comparing the numerical results with experiments. The effects of manufacturing are described for the six prototypes using CFD simulation. The implementation of the friction loss model, the affinity law model, and the lift height model used in the case study will be presented below.

### Validation of the CFD approach

To validate the CFD approach and to compare field experiments with CFD simulation results, impellers 3 and 4 were tested physically and simulated numerically. The results are shown in Table 1. The results demonstrate consistency between the CFD simulation and the field experiments, as both shut‐off head and flow rate exhibit small percentage differences within 8% of the desired operating range (1500 and 1744 RPM). A pump speed of 1000 RPM serves as a lower boundary of the test but is not a typical operating shaft speed for MFP according to specific speed analysis. Note that because the CFD simulation only tests the performance of the pump segment, it neglects the friction losses of the other components in the system. Therefore, the flow rate predictions are much greater than the experimentally measured flow rates. As a result, it is necessary to scale down the simulation's flow rates based on friction loss analysis to match the experimental results.

**Table 1:** Comparison of numerical simulation and field testing results

Impeller number	RPM	From simulation		From full‐scale testing		% Difference	
		Shut‐off head (m)	Flow rate after scaling (l s^‐1^)	Shut‐off head (m)	Flow rate (l s^‐1^)	Shut‐off head (m)	Flow rate (l s^‐1^)
3	1744	9.04	48.9	8.37	47.9	8.0	2.1
	1500	6.89	42.1	6.59	42.5	4.6	1.0
	1000	3.01	26.0	2.02	21.8	49.0	19.0
4	1744	8.72	49.2	8.74	48.4	0.2	1.7
	1500	6.45	43.2	5.98	40.3	7.9	7.3
	1000	2.85	26.7	2.02	21.9	41.1	21.9

Note: % Difference = (Full‐scale Testing – Simulation) /Full‐scale testing.

### Geometric variation effects

The results of CFD simulations corresponding to geometric variations due to manufacturing for the six impeller prototypes at 1500 and 1744 RPM are shown in Table 2. The sample standard deviations of the shut‐off head generated by the six impellers at 1744 and 1500 RPM are 0.27 and 0.29 m, which correspond to 3.11 and 4.43% of the average head. These small values demonstrate that geometric variations due to manufacturing have little effect on performance.

**Table 2:** Effect of geometric variation due to manufacturing on performance evaluated by shut‐off head (m)

Impeller number	RPMs	
	1744	1500
1	8.57	6.06
2	8.60	6.76
3	9.04	6.89
4	8.72	6.45
5	8.28	6.65
6	8.94	6.52
Average (AVE)	8.69	6.55
Standard deviation (STD)	0.27	0.29
STD/AVE	3.11%	4.43%

### Friction loss model

The concept of major and minor losses was introduced above to estimate pump performance of the components, and thus their friction losses. The loss coefficients for each component within the system are identified in Tables 3 and 4 and are estimated from their material and geometry (Crane Co. Engineering Division 2012). The sum of the major loss coefficients is 3.94, and the sum of the minor loss coefficient is 13.2. This results in a total loss coefficient of 17.2.

**Table 3:** Major losses associated with full‐scale testing apparatus

No.	Pipe material	Major loss coefficient, $K_{\text{major}}=f\frac{l}{D}$
1	GI pipe with inner shaft	1.22
2	Flexible pipe	2.43
3	Worn cast iron	0.29
	Total major losses, *K*_major_	3.94

**Table 4:** Minor losses and total losses associated with full‐scale testing apparatus

No.	Feature	Minor loss coefficient, *K*_minor_
1	Inlet cage	2.62
2	Diffuse vane	1.08
3	Protruding pipe entrance	0.80
4	Gradual contraction	0.26
5	Shaft bearing	1.08
6	Wye flow	0.32
7	Crossing shaft	0.005
8	Pressure fitting 1	0.80
9	Pressure fitting 2	1.00
10	Woltman flowmeter WPH‐150	1.43
11	Tee threaded dividing line flow	0.90
12	Ball valve	0.05
13	Pressure gauge	0.27
14	Butterfly valve	1.62
15	Sharp‐edged exit	1.00
	Total minor losses, *K*_minor_	13.2
Sum of minor and major losses, *K = K*_major_ *+ K*_minor_		17.2

The calculated loss coefficient is obtained by operating prototypes 1, 3 and 4 at different RPMs, and then using Equation (5). The sum of the major and minor loss coefficients has an average of 21.8 ±1.7 SD (Yu 2017).

### Affinity law model tests

**Figure 6:**
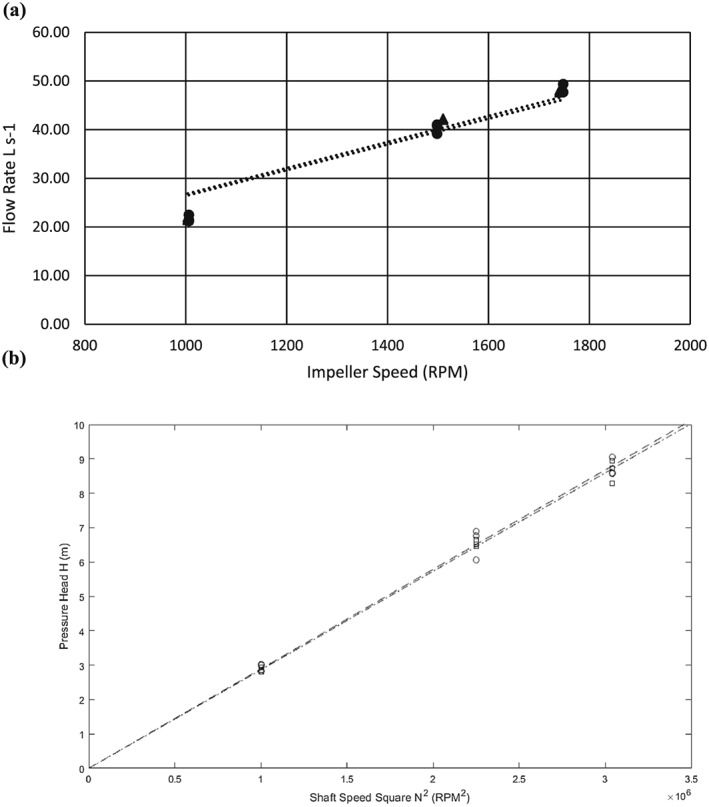
Affinity law evaluation of (a) experimental data, and (b) numerical data. Impeller 3 (▲), impeller 4 (●). Linear curve fits: impeller 3: *y* = 0.0268 *x*, *R* ^2^ = 0.92; impeller 4: *y* = 0.264 *x*, *R* ^2^ = 0.92.

Lines are fitted through the origin of experimental and simulation results to correlate pump rotational speeds and discharge rates (Figure 6). Both results demonstrate good agreement with the affinity law, as shown by *R* ^2^ values above 0.9 in all four cases.

### Lift height model

**Figure 7:**
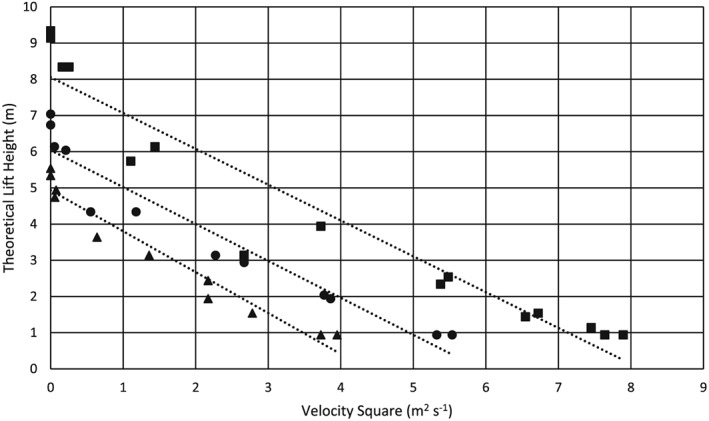
Theoretical lift height versus velocity squared. ▲ = 1498 RPM, ● = 1740 RPM, ■ = 2065 RPM. Linear curve fits: 1498 RPM: *y* = −1.31 *x* + 4.93, *R* ^2^ = 0.95; 1740 RPM: *y* = −1.02 *x* + 6.04, *R* ^2^ = 0.93; 2065 RPM: *y* = −0.989 *x* + 8.06, *R* ^2^ = 0.92.

The lift height model described above is plotted in Figure 7. After fitting the data using linear regression, the model is validated by high *R* ^2^ values of 0.95, 0.93 and 0.92 when operated at 1489, 1740 and 2065 RPM, respectively.

## Discussion

The comparison of CFD simulations and experimental results demonstrates that the impellers have a steadier behaviour at higher shaft speeds (Table 1). At 1500 and 1744 RPM, both impellers have differences between simulation and experiments of less than 8% for both shut‐off head and flow rate. At 1000 RPM, this difference rapidly increases to approximately 22% in flow rate and over 41% in shut‐off head. This validates the CFD model at 1500 and 1744 RPM, but not at 1000 RPM. A possible source of this mismatch is open‐channel flow seen in field testing at 1000 RPM. At this speed, the pump barely lifts the water above the level of the exit, leaving the pipe partially filled with water. This results in an open‐channel flow, which affects the results.

In practice, however, this non‐ideal behaviour at low shaft rotational speeds is of little consequence because AFPs and MFPs are most efficient at high rotational speeds (Santos Valle *et al*. 2014). At low shaft speeds, such as 1000 RPM, centrifugal pumps are likely to have greater efficiency. It can be concluded that the results from numerical simulation agree well with the results of the field testing in the desired range of operation. The effect of geometric variation due to manufacturing was therefore tested at high rotational speeds using the CFD model as described below.

The small standard deviations of pump performance at the two higher shaft speeds demonstrates the limited effect of dimensional inaccuracies due to manufacturing on pump performance as evaluated by the shut‐off head. Based on this result, our partner pump manufacturer in Bangladesh can be confident that their MFP impellers can generate heads of 5.6–7.5 m and 7.8–9.6 m at pump speeds of 1500 and 1744 RPM, respectively, for the current pump tested. This is a crucial result because domestic manufacturing of AFPs and MFPs in Bangladesh is now growing to commercial scales (CSISA‐MI 2016). This consistency in shut‐off head indicates a small manufacturing effect on the discharge rate, which could be further improved for better pump performance.

**Figure 8:**
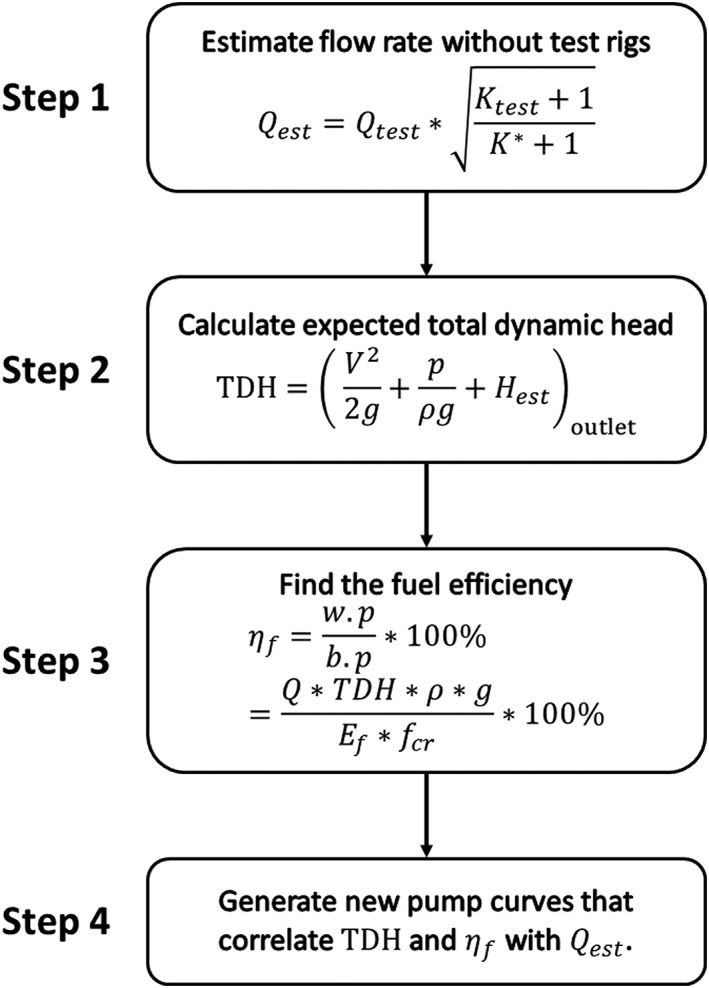
Flow chart for method of pump selection.

Using these results, a method of pump selection can be developed based on fuel efficiency at specified operating conditions through the following four steps (Figure 8).

Step 1 eliminates the effect of test rigs and thereby estimates the discharge rate without physically testing the devices. Based on the friction loss model, the performance of pumping systems with customized components (without test rigs) can be estimated using Equation (11) from results of a system tested at the same shaft speed and lift height:

(11) $$Q^{*}=Q_{\text{test}}*\sqrt{\frac{K_{\text{test}}+1}{K^{*}+1}}$$

where *Q* ^*^ is estimated discharge of the new system, *Q*_test_ is the discharge from experimental results of the tested system, *K*_test_ is the loss coefficient of the tested system and *K* ^*^ is the estimated loss coefficient of the new system.

Step 2 calculates the expected total dynamic head (TDH) using the estimated discharge rate. By definition, expected TDH is calculated from two end points of a continuous streamline, which are the surface level of the water source (with approximately zero velocity, gauge pressure and elevation) and the pipe outlet (Karassik *et al*. 2007). This simplifies to Equation (12):

(12) $$\mathrm{TDH}=\Delta \left(\frac{V^{2}}{2g}+\frac{p}{\mathrm{\rho g}}+H\right)={\left(\;\frac{V^{2}}{2g}+\frac{p}{\mathrm{\rho g}}+H_{\mathrm{est}}\right)}_{\text{outlet}}$$

where $V=Q/\left(\pi d^{2}/4\right)$ is the average velocity. Note that due to the friction loss being directly proportional to *V* ^2^, TDH decreases as flow rate increases.

Step 3 calculates the fuel efficiency as described by Equation (13):

(13) $$\eta_{f}=\frac{w.p}{b.p}*100\%=\frac{Q*\mathrm{TDH}*\rho *g}{E_{f}*f_{\mathrm{cr}}}*100\%$$

where *f*_cr_ is the fuel consumption rate measured from physical pump tests and *E*_f_ the fuel energy content (35.9 × 10^6^ J l^‐1^ for diesel) (Edwards *et al*. 2011). Step 4 generates curves that correlate expected TDH and fuel efficiency with flow rate for a pump running at a fixed rotational speed.

**Figure 9:**
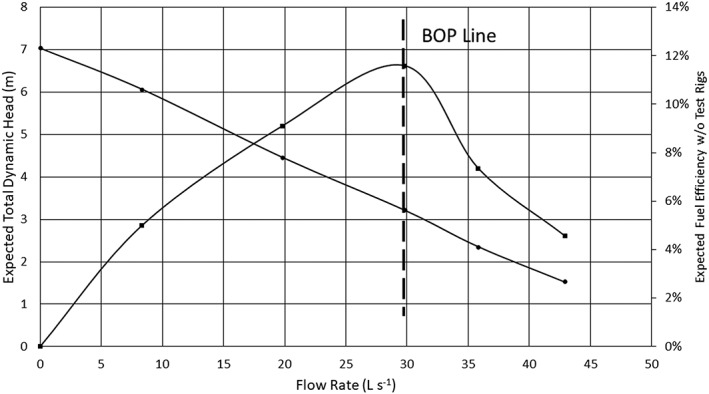
Sample curve for expected TDH and fuel efficiency versus flow rate, with best operation point (BOP). ● = TDH 1740 RPM; ■ = Efficiency 1740 RPM.

This performance evaluation method is not limited to MFPs but is also applicable to axial flow and centrifugal pumps. In Figure 9, a sample curve is generated for prototype 1 at 1740 RPM. In the figure, the best operation point (BOP) is found at the point of highest fuel efficiency, approximately 30 l s^‐1^. Similar curves can be generated for other pumps and operating conditions, which again can simply the process of design and pump manufacturing where fuel efficiency is an important goal, as is the case with many axial‐ and mixed‐flow pump tests (Krupnik *et al*. 2015; Santos Valle *et al*. 2014; Stickney and Salazar 1989; Chinsuwan and Cochran 1986; Aban 1985).

**Figure 10:**
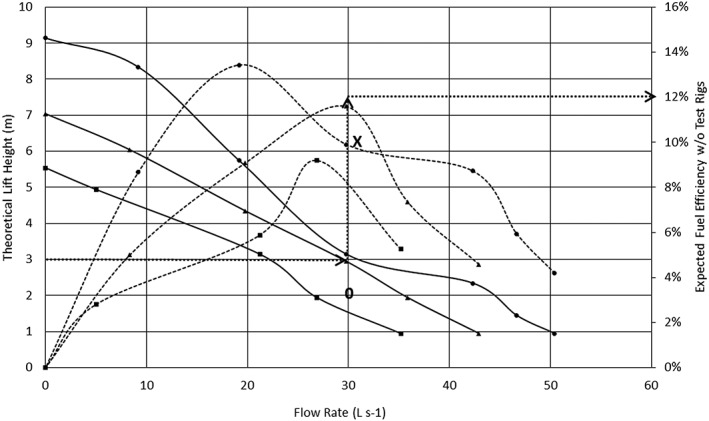
New pump curve for pump selection. Note that the expected total dynamic head is renamed here as the theoretical lift height, which may be better understood by less technically inclined audiences. ■ = 1498 RPM; ▲ = 1740 RPM; ● = 2065 RPM. Solid lines = lift height; dashed lines = efficiency.

This approach is likely to be more straightforward, effective, expedient and simpler for pump selection than traditional pump curves, although it does require careful data measurements and knowledge of modelling concepts and fluid dynamics (Lam *et al*. 2017), which may require capacity‐building efforts in some country contexts. To demonstrate these concepts, we derived a sample figure using the data set shown in Figure 10. In this case, pump performance at various rotational speeds is plotted in combination.

These concepts are best understood with a practical example. Typically, farmers interested in purchasing pumps are likely to consider whether a specific pump meets their expectations for lift height, flow rate, cost and fuel efficiency. Using Figure 10 or similarly derived graphs, farmers, irrigation pump service providers and agricultural machinery manufacturers and dealers can select a desired pump speed from the theoretical lift height curve. Expected efficiency can also be located on the efficiency curve at the same flow rate. An example is shown by dotted arrows for a pump owner desiring a lift height of 3 m and a flow rate of 30 l s^–1^. In this situation, this chart can be used to obtain a better operating shaft speed at approximately 1750 to 2000 RPM for a higher fuel efficiency of 12% as opposed to 10%, as denoted by the data point to the left of the ‘X’ on the figure.

In practice, farmers may not be able to obtain the performance data at shaft speeds described in this figure. In this case, the solution may be approximated using the affinity law model discussed above to scale pump performance from that of the closest tested shaft speed. For example, if one desires a lift height of 2 m and a same flow rate of 30 l s^–1^, then one can select that flow rate at approximately 1/3 between 1498 and 1740 RPM, indicated by the ‘0’ in Figure 10. As the pump speed is directly proportional to the flow rate, this indicates the most fuel efficient operation at 1579 RPM, as shown in Equation (14):

(14) $$1498+\frac{1740-1498}{3}=1579\;\mathrm{RPM}$$

This process facilitates determining the fuel efficiency of different pumps, and thus can be useful in terms of selecting ‘best‐bet’ pump types considering economic and energy efficiency goals. Pump selection using similarly derived efficiency curves has three major advantages compared to a traditional pump curve (Henshaw 2017). First, the new pump curve is more organized and simpler to plot. The multiple efficiency curves on a traditional pump curve are also avoided, providing increased ease of interpretation, especially for less technically oriented audiences. Second, these discrete efficiency curves are replaced by a linear curve in the new approach, which allows users to compare efficiency at multiple operating conditions more easily. Finally, the new pump curve plots the performance of various pump speeds consecutively. As opposed to the traditional approach that involves the manipulation of multiple pump curves, this refined approach facilitates the more intuitive determination of the best operating speed from one chart with less effort. Note, however, that this new pump curve is generated for the same pump type at different operating conditions; to compare pumps of different types or diameters, multiple pump curves would be required.

When implementing this simplified pump testing method, the user should be aware of several limitations. First, this method is only valid within the desired operating range of axial‐ and mixed‐flow pumps. It should not be applied at low operating speeds (<1000 RPM). Second, this method does not incorporate ageing or life‐cycle analysis of pumping equipment. Third, this method lacks performance assessments by end users. In the future, this simplified pump testing method is expected to be further tested with manufactured samples used by local farmers to evaluate its effectiveness. In addition, this method could also be modified to facilitate centrifugal pump testing.

## Conclusions

This paper proposes a pump testing approach for AFPs and MFPs used for crop irrigation in resource‐limited developing countries. Through a case study in Bangladesh, this study investigates the local manufacturing capability. By testing a set of six impeller prototypes manufactured in Bangladesh, it observes that the standard deviation of performance within the set is below 5% of average values, and thus it concludes that manufacturing differences among prototypes have little effect on their performance and ensures the manufacturability of the product.

The affinity law, a friction loss model, and a lift height model are incorporated into the analysis and validated with full‐scale field testing. Based upon these models, an innovative pump selection method is introduced to interpret and compare pump performance. Compared to traditional pump curves, this approach is more straightforward, effective and simpler to use for local customers, service providers and sales personnel, allowing them to easily find the most economic and energy‐efficient solution. Finally, the outcomes of the pump selection method and the manufacturing capability are both validated with CFD modelling. Currently, pumps tested with the proposed method are fabricated and sold in Bangladesh, increasing energy efficiency up to 50% from that of CPs at low operating heads.
